# Spectromicroscopic insights for rational design of redox-based memristive devices

**DOI:** 10.1038/ncomms9610

**Published:** 2015-10-19

**Authors:** Christoph Baeumer, Christoph Schmitz, Amr H. H. Ramadan, Hongchu Du, Katharina Skaja, Vitaliy Feyer, Philipp Müller, Benedikt Arndt, Chun-Lin Jia, Joachim Mayer, Roger A. De Souza, Claus Michael Schneider, Rainer Waser, Regina Dittmann

**Affiliations:** 1 Peter Gruenberg Institute, Forschungszentrum Juelich GmbH and JARA-FIT, 52425 Juelich, Germany; 2 Institute of Physical Chemistry, RWTH Aachen University and JARA-FIT, 52056 Aachen, Germany; 3 Ernst Ruska-Centre, Forschungszentrum Juelich GmbH and RWTH Aachen University, 52425 Juelich, Germany; 4 Institute of Materials in Electrical Engineering and Information Technology II, RWTH Aachen University, 52056 Aachen, Germany

## Abstract

The demand for highly scalable, low-power devices for data storage and logic operations is strongly stimulating research into resistive switching as a novel concept for future non-volatile memory devices. To meet technological requirements, it is imperative to have a set of material design rules based on fundamental material physics, but deriving such rules is proving challenging. Here, we elucidate both switching mechanism and failure mechanism in the valence-change model material SrTiO_3_, and on this basis we derive a design rule for failure-resistant devices. Spectromicroscopy reveals that the resistance change during device operation and failure is indeed caused by nanoscale oxygen migration resulting in localized valence changes between Ti^4+^ and Ti^3+^. While fast reoxidation typically results in retention failure in SrTiO_3_, local phase separation within the switching filament stabilizes the retention. Mimicking this phase separation by intentionally introducing retention-stabilization layers with slow oxygen transport improves retention times considerably.

## Abstract (web summary)

Memristive devices offer a future low-power solution to data storage and logic operations, but there is still a lack of suitable material design rules. Here, the authors present a design rule for retention-failure-resistant devices based on spectromicroscopic studies of strontium titanate.

## Graphical abstract

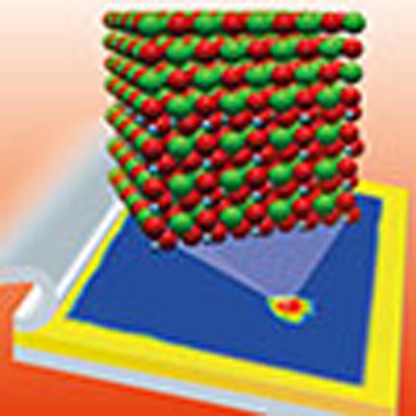

Driven by the technological need for novel materials and innovative device concepts for future data storage and logic circuits, researchers in industry and academia are investigating intensively memristive transition metal oxides. This interest is due to the materials' potential use in future non-volatile memory or as novel neuromorphic circuits, even allowing for a local synergy of logic and memory^1^,^2^,^3^,^4^,^5^,^6^. The mechanism of resistive switching in transition metal oxides is widely accepted to be a nanoscale redox reaction, induced by oxygen-ion migration, the so-called valence-change mechanism^7^,^8^,^9^,^10^,^11^,^12^. During the SET processes, oxygen vacancies, which act as donor dopants and contribute electrons into the conduction band, form a so-called switching filament. The resulting local dopant distribution modifies the Schottky barrier at the interface with the top electrode and leads to a lower device resistance, the low-resistance state (LRS). RESET operations with opposite bias recover the high-resistance state (HRS) by disrupting or reoxidizing the switching filament^13^. Many research groups in academia and industry have already presented devices with a comparably simple architecture and excellent device performance. The ultimate aim regarding device performance is high uniformity for all devices on a single chip, an endurance (write cyclability) of at least 10^7^ cycles, scalability down to the nanometre regime and the potential for multibit operation^3^,^5^. Most of all, the ultra-nonlinear switching kinetics (also called voltage–time dilemma) between extremely fast switching times (≤10–100 ns) and long retention times (exceeding 10 years) has to be met for non-volatile memory applications^3^.

While excellent retention has been demonstrated for many binary and ternary oxides in conjunction with fast switching, Noman *et al.*^14^ showed that in the absence of internal electric fields, the model valence-change material SrTiO_3_ cannot exhibit both properties simultaneously. In fact, retention failure after short times was reported for the LRS in single-crystalline SrTiO_3_ (refs 15, 16, 17). Polycrystalline and single-crystalline SrTiO_3_ films with considerable amounts of extended defects, on the other hand, exhibit much better retention behaviour^16^,^18^. On the basis of simulations of the *I*–*V* characteristics and retention times, one finds that the retention failure mechanism for the LRS is based on the rupture of conducting filaments caused by reoxidation due to oxygen diffusion from the side^19^,^20^ or along the vertical direction^21^. Recent studies on the technologically most relevant systems of HfO_2−*x*_ (refs 22, 23, 24, 25, 26) and Ta_2_O_5−*x*_ (refs 19, 20, 27, 28), however, reveal that extremely high retention times can be achieved with oxidizable electrodes or interlayers (the so-called oxygen-scavenging layer). Phenomenologically, this finding was attributed to the stability of certain oxygen distributions in the layer stack and filaments with sufficient oxygen-vacancy concentrations to be stable against reoxidation due to oxygen diffusion in the lateral direction. Experimental evidence for the role of local redox reactions for both the device function (resistance change between LRS and HRS) and for the retention failure, however, remains elusive so far, while some qualitative conclusions from these simulation-based studies^20^,^21^,^29^ such as the importance of the filament size and the oxygen-vacancy concentration within the filament and the adjacent layers certainly hold true.

Here we will experimentally demonstrate that one decisive (but previously overlooked) factor for the retention of memristive devices is the presence or formation of an oxygen-migration-blocking layer. We will show that for a retention-failure-resistant valence change in the model material SrTiO_3_, a local phase separation into a switching element and an artificial retention-stabilization layer is necessary to obtain the required nonlinearity of the kinetics. Single-crystalline SrTiO_3_ was chosen as the model material due to its homogeneity, the absence of grain boundaries as well as the extensive existing knowledge about defect chemistry, oxygen diffusion and cation diffusion available in literature^30^,^31^,^32^,^33^,^34^,^35^,^36^. Using spectromicroscopy, we explicitly confirm that the resistance changes that occur during device operation and retention failure are caused by oxygen migration and corresponding localized redox reactions between Ti^3+^ and Ti^4+^ configurations. We use these insights to demonstrate a new design rule for improved retention times in SrTiO_3_ devices: the intentional insertion of a retention-stabilization layer. The crucial parameter of the retention-stabilization layer is its capability to impede oxygen migration, thus protecting the switching filament from reoxidation. This finding paves the way for a rational design of reliable memristive cells for future memory or logic applications through the judicious choice of the oxygen-scavenging layer or intentional retention-stabilization layer.

## Results

### Spectromicroscopic analysis of resistance changes in SrTiO_3_

**Figure 1:**
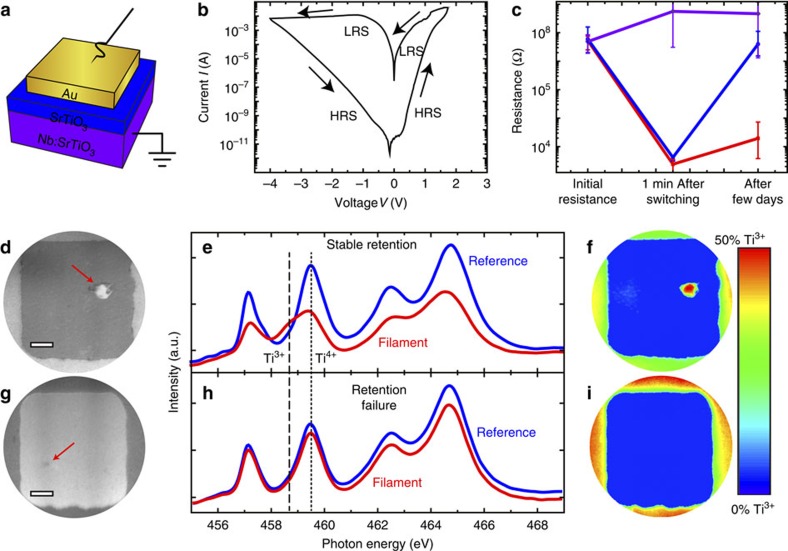
Ti L-edge XPEEM analysis of SrTiO_3_ memristive devices. (**a**) Device schematic. (**b**) Representative *I*–*V* curve. (**c**) Retention behaviour of different devices on the same chip. Devices RESET into the HRS remain at a constant resistance over several days (violet curve). Devices that were SET into the LRS show two classes of retention behaviour: stable retention and retention failure (red and blue curves, respectively). For each class of retention behaviour, the resistance was averaged for five representative devices. Error bars indicate the minimum and maximum values obtained for each resistance state. (**d**) XPEEM image of a device with a stable retention recorded with a photon energy of 458.5 eV. Filament indicated by a red arrow. Scale bar, 2 μm. (**e**) Ti L-edge spectra extracted from the XPEEM image stack for a region inside the bright filament discernable in **d** (red line) and for the surrounding device area (blue line). The dotted line and the dashed line denote the *e*_g_ level of the Ti L_3_ edge in Ti^4+^ and Ti^3+^ configuration, respectively. (**f**) False colour map of the Ti^3+^ contribution for the device in **d**. Only within the suspected switching filament, there is a significant spectral contribution of Ti^3+^. The enhanced contrast surrounding the active device is caused by the reactive ion beam etching involved in the electrode structuring and is not associated with the device conductance. (**g**) XPEEM image of a device with retention failure recorded with a photon energy of 459.5 eV. (**h**) Ti L-edge spectra extracted from the XPEEM image stack for a region inside the suspected switching filament discernable in **g** (red line) and for the surrounding device area (blue line). (**i**) False colour map of the Ti^3+^ spectral contribution for the device in **g**. No Ti^3+^ contribution is discernible for the entire device area.

To visualize switching-induced local changes of the electronic and chemical structures of SrTiO_3_, we investigated memristive Au/SrTiO_3_ (2 nm)/Nb:SrTiO_3_ devices (Fig. 1a) using X-ray photoemission electron microscopy (XPEEM, see the Methods section for fabrication details). For this purpose, multiple devices on the same chip were switched between the HRS (∼10^7^–10^8^ Ω) and the LRS (∼10^3^–10^4^ Ω) several times using a current compliance of 50 mA as shown in Fig. 1b. No separate forming step was necessary: the first application of positive voltages (SET process) led to a so-called soft-forming, which was very similar to subsequent SET processes. The resistance of each device was monitored over a period of several days (Fig. 1c). While the HRS was stable for each device investigated here, two classes of retention characteristics of the LRS were observed: for most devices in the LRS, the resistance increased by several orders of magnitude, indicative of a retention failure, while some devices (∼20%) retained a resistance below 10^5^ Ω. In the following, these devices will be referred to as devices with a retention failure and devices with stable retention, respectively.

In a first step, the devices with stable retention were used to examine the valence-change mechanism, which is expected to be responsible for the resistive switching in SrTiO_3_. After delamination of the top electrode (see Methods), XPEEM imaging of a representative device with stable retention revealed a switching filament with enhanced contrast (Fig. 1d). As the photon energy *h*ν=458.5 eV used in this case corresponds to the Ti L_3_ absorption edge, one may surmise that the contrast arises because of a reduced Ti valence state inside this filament, which has been expected to be the origin of the low resistance^3^,^9^,^11^,^37^,^38^. In fact, extracting Ti L-edge absorption spectra for a region of interest within this filament and for a reference area from the surrounding reveals a strong contribution of Ti^3+^ within the filament, indicated by the dashed line, while the surrounding device area only shows Ti^4+^ contributions, as is expected for stoichiometric SrTiO_3_ (Fig. 1e)^11^,^39^. The local confinement of this valence change is evident from the false colour map of the Ti^3+^ contribution for the entire device area as shown in Fig. 1f: only for the filament area does one find a significant contribution. Since Ti^3+^ states in SrTiO_3_ correspond to electrons in the conduction band^3^, this region can in fact be regarded as the switching filament. We thus conclude that the resistive switching occurs within this filament due to a local reduction of the Ti.

While a locally confined, chemically reduced component has been previously observed after electroforming^7^,^10^,^11^,^40^,^41^ for different oxide materials, the different valence states between LRS and HRS have not been demonstrated until now. We therefore show that the observed Ti^3+^ valence change in our samples is reversible: for a device that was switched back to the HRS, no Ti^3+^ spectral contributions could be detected (Supplementary Fig. 1) although XPEEM images indicate the presence of a locally modified area, which we ascribe to the reoxidized switching filament. In other words, we have demonstrated explicitly that field-driven local reduction and reoxidation indeed constitute the fundamental mechanism behind resistive switching in this model system.

As discussed in the introduction, reoxidation of the switching filament, albeit on a different (longer) timescale, is also believed to be responsible for the retention failure of the LRS. We analysed, therefore, the local electronic structure of devices with retention failure, as shown for an exemplary device in Fig. 1g–i. While the XPEEM images show a small area with a darker contrast, which might be the remainder of a switching filament, the Ti L-edge absorption spectra for this region and the surroundings, and in particular the false colour map of Ti^3+^, reveal that no significant Ti^3+^ contribution is present in the entire device area. As the SrTiO_3_ film is only 2 nm thin, which is significantly smaller than the probing depth of our XPEEM measurements (∼5 nm), this means that the initially reduced switching filament is completely reoxidized during retention failure. The role of oxygen migration for the retention of SrTiO_3_-based devices is also directly apparent from the comparison of the retention characteristics in vacuum with the behaviour in ambient atmosphere (Supplementary Fig. 2). The memory window (ratio between HRS and LRS) after 5 × 10^5^ s in vacuum is one order of magnitude higher than for samples kept in air. The retention times are improved because no oxygen is available for reoxidation of the switching filament from the environment. Consequently, these results experimentally verify the hypotheses of previous simulation-based models^21^ that propose the retention failure of the LRS to be due to oxygen diffusion along the vertical direction. Given that the reoxidation is inhibited in vacuum, oxygen species necessary for retention failure are evidently not supplied from the lateral direction. Instead, the oxygen species are apparently stored at the interface between the top electrode and the SrTiO_3_ film, inside the top electrode (for example, within grain boundaries), or even supplied from the surrounding atmosphere. We can thus conclude that incorporation and vertical diffusion of oxygen from the top electrode or surrounding into SrTiO_3_ plays a dominant role in the retention-failure mechanism.

**Figure 2:**
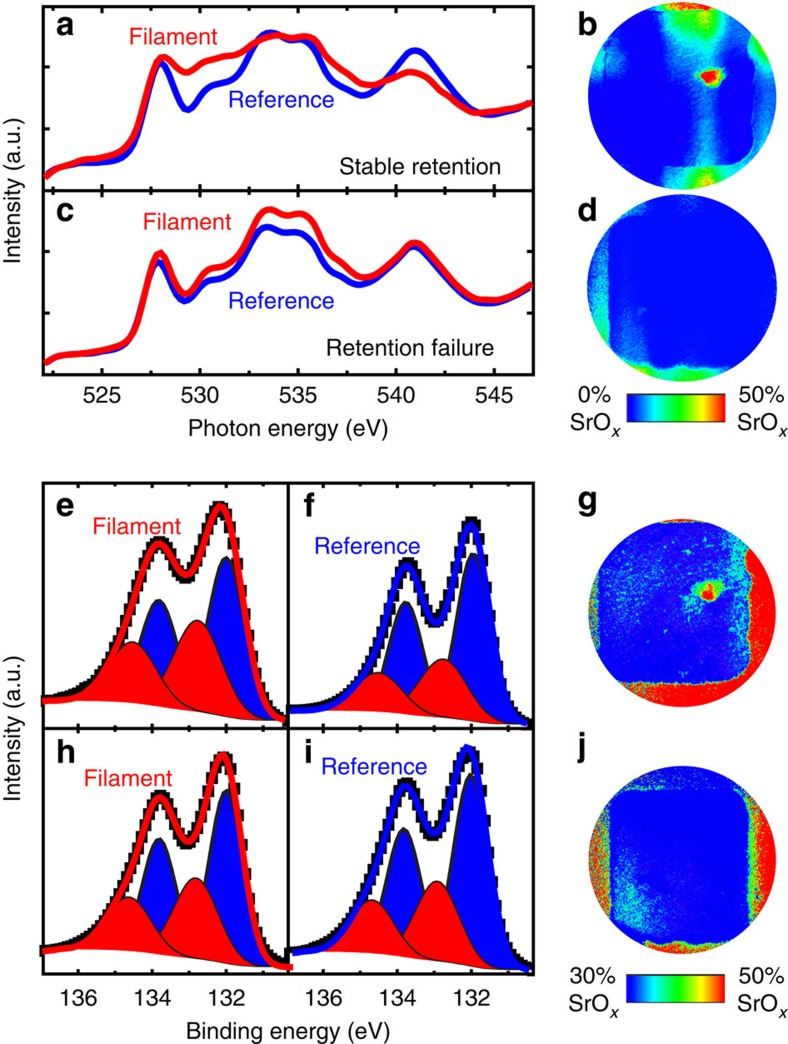
O -K-edge and Sr 3d XPEEM analysis of SrTiO_3_ memristive devices. (**a**) O-K-edge spectra extracted from the XPEEM image stack for the switching filament (red line) and for the surrounding device area (blue line) for the device with stable retention (the same device as in Fig. 1d–f). (**b**) False colour map of the SrO contribution for the same device. Only within the suspected switching filament, there is a significant spectral contribution of SrO. The apparent vertical stripe in the centre of the device is an artefact from the uncorrected but small horizontal energy dispersion of the NanoESCA energy filter. The resulting contrast is much lower, however, than the real contrast detected within the filament. (**c**) O-K-edge spectra extracted from the XPEEM image stack for a region within the switching filament (red line) and for the surrounding device area (blue line) for the device with retention failure (the same device as in Fig. 1g–i). (**d**) False colour map of the SrO contribution for the same device. No significant contribution of SrO can be detected. (**e**) Sr 3d spectrum for the switching filament (black data points) for the device with stable retention with a fit (red line) consisting of a bulk component (blue doublet) and a SrO-like surface component (red doublet). (**f**) Sr 3d spectrum extracted from a region surrounding the switching filament (black data points) for the same device with a similar fit (blue line). The SrO-like surface component is suppressed in comparison with **e**. (**g**) False colour map of the SrO contribution for the same device. Only within the suspected switching filament, there is a significant contribution of SrO. (**h**) Sr 3d spectrum for the suspected switching filament (black data points) for the device with retention failure with a similar fit (red line). (**i**) Sr 3d spectrum extracted from a region surrounding the switching filament (black data points) for the same device and a similar fit (blue line). The SrO-like surface component is equally small in both spectra. (**j**) False colour map of the SrO contribution for the same device. No significant contribution of SrO can be detected.

As the reoxidation of switching filaments evidently needs to be inhibited to protect a device from retention failure, the question arises why some devices on the same chip show stable retention, while most devices exhibit retention failure. Given the comparably fast ambipolar diffusion of oxygen vacancies in SrTiO_3_ (refs 30, 31, 32), we would predict retention failure for every device^14^. To elucidate the microscopic mechanism for retention stabilization, we carefully analysed the local chemical configuration of the switching filaments of the devices shown in Fig. 1. O -K-edge absorption spectra for the filament and the surroundings of the device with stable retention reveal a strong modification of the local environment of the O-ions within the filament (Fig. 2a). From comparison with literature, we assume that this modification stems from a significant SrO contribution^42^, which is plausible as the formation of SrO islands on the surface of SrTiO_3_ has been previously observed after high-temperature treatments exceeding 1,000 °C (refs 43, 44, 45). It has already been shown that Joule heating during electrical treatment can provide sufficiently high temperatures to promote such a phase separation^9^,^11^,^46^. The false colour map of the SrO contribution to the O -K-edge shown in Fig. 2b reveals that this modified O-environment is only present within the switching filament for the device with stable retention and the device in the HRS (Supplementary Fig. 1), while it is absent for the device with retention failure (Fig. 2c,d). We thus propose that in the first case, significant Joule heating within the filament leads to a localized phase separation into a Sr-deficient SrTiO_3_ and a SrO interface component, which becomes a surface component after delamination. As SrO surface components have been detected using X-ray photoelectron spectroscopy (XPS)^46^,^47^ before, we performed energy-resolved XPEEM for both devices for the Sr 3d level to verify the anticipated phase separation. As typically observed for SrTiO_3_ thin films, the Sr 3d spectra extracted from these scans can be fitted well with a doublet for the bulk contribution and an additional doublet found at 0.8 eV higher binding energy, which corresponds to SrO-type surface components (Fig. 2e,f)^46^,^47^. As expected, the amount of this surface component is significantly higher within the filament of the device with stable retention, as is evident from the representative spectra for the filament and the surrounding and the false colour map of the SrO contribution shown in Fig. 2e–g. The electron kinetic energy used here was around 60–70 eV, corresponding to the minimum of the kinetic-energy-dependent inelastic mean free path of just 0.5 nm (ref. 48). The resulting surface sensitivity of the XPS analysis thus supports the hypothesis that the observed phase separation for devices with stable retention yields a Sr-deficient filament with a SrO layer in the surface region.

As for the O -K-edge, there is no indication of this local phase separation in the XPS analysis for the device with retention failure (Fig. 2h–j). Consequently, it appears that the phase separation into a Sr-deficient SrTiO_3_ and a SrO surface layer is mandatory for stable retention. This is further corroborated by a comparison of all measured devices: four devices with retention failure and four devices with stable retention were investigated with XPEEM. For all of these devices, we observed no Ti^3+^ or SrO spectral contribution for the devices with retention failure, while all devices with stable retention exhibited a filament with a Ti^3+^ contribution and an SrO interface component. Intuitively, this phase separation could be presumed to be responsible for strongly suppressed reoxidation of the switching filament from the vertical direction due to impeded oxygen diffusion within and through the SrO layer. Reoxidation from the side is unlikely to contribute significantly due to the filament dimensions, which would require μm diffusion lengths from the side in contrast to only 2 nm in the vertical direction. The diffusion of oxygen in SrO, however, is not well understood. A theoretical description of oxygen migration in this system will therefore be provided below. Beforehand, we will have to consider the question of whether the observed phase separation is a side effect of the process leading to stable retention, or indeed its origin. To demonstrate and make use of the influence of the SrTiO_3_/SrO interface for the device performance, we investigated the retention characteristic of an intentionally created interface between Sr-deficient SrTiO_3_ and SrO.

### Stable retention behaviour in SrTiO_3_/SrO heterostructures

**Figure 3:**
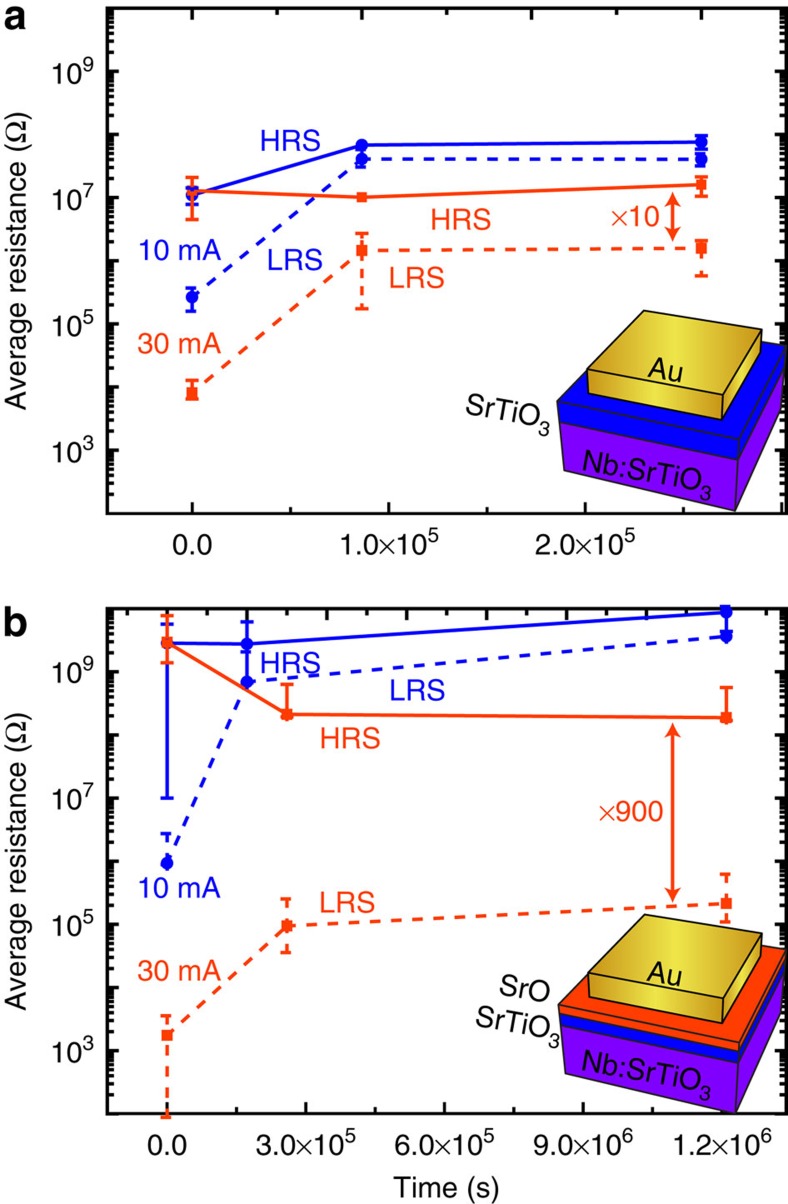
Retention behaviour of SrTiO_3_ thin-film devices and SrTiO_3_/SrO heterostructures. (**a**) Average resistance of SrTiO_3_ thin-film devices in the LRS (dashed line) and the HRS (solid line) as a function of time after switching for a SET current compliance of 10 mA (blue lines) and 30 mA (red lines). The device schematic is shown in the inset. (**b**) Average resistance of SrTiO_3_/SrO heterostructure devices in the LRS (dashed line) and the HRS (solid line) as a function of time after switching for a SET current compliance of 10 mA (blue lines) and 30 mA (red lines). Error bars indicate the minimum and maximum values obtained for each resistance state. The device schematic is shown in the inset.

To mimic the phase separation described above, artificial SrTiO_3_/SrO heterostructures were created by successive pulsed laser deposition of 2 nm ∼1 at% Sr-deficient SrTiO_3_ and 1 nm SrO (ref. 49). For the quantitative comparison of the retention behaviour of SrTiO_3_ thin-film devices as investigated in Fig. 1 with SrTiO_3_/SrO heterostructures, multiple devices were switched between the HRS and LRS configurations for both samples and the resistance values were monitored over several days. To reveal the retention behaviour of the as-prepared films rather than the characteristics of local phase separations as described above, the current compliances were chosen as low as possible such that the memory window was still larger than two orders of magnitude^5^,^19^. The *I*–*V* hysteresis for both samples remained similar in shape, indicating that the same fundamental switching mechanism was responsible for the resistance change (Supplementary Fig. 3). The average resistance as a function of current compliance and time is shown in Fig. 3a,b. While the retention of the HRS was rather stable for each case, the LRS showed strong differences between both samples, depending on the current compliance. For 10 mA, the average LRS increased to a value barely distinguishable from the HRS within a short time for both SrTiO_3_ thin-film devices and SrTiO_3_/SrO heterostructures. For 30 mA, on the other hand, LRS and HRS are also very close to each other for SrTiO_3_ thin-film devices after 10^5^ s (or even earlier), but SrTiO_3_/SrO heterostructures exhibit a large memory window of roughly three orders of magnitude even after 10^6^ s. While the data shown in Fig. 3 is averaged over multiple devices, we would like to note that in contrast to the SrTiO_3_ thin-film devices, we did not observe any device with a complete retention failure (HRS and LRS indistinguishable) for the SrTiO_3_/SrO heterostructures after switching with 30 mA. This improved retention behaviour is caused by a rather stable LRS value after an initial relaxation combined with a stable HRS on the order of 10^8^ Ω. It was achieved with less power than was necessary for the observation of single-SrTiO_3_ devices with stable retention (cf. Fig. 1), making it less likely that an additional phase separation is responsible for the stable retention. The dispensability of phase separations for good retention times also results in a more uniform behaviour of different devices on the same chip: while the devices shown in Figs 1 and 2 are vastly different in their retention behaviour, all devices containing the intentional SrTiO_3_/SrO interfaces exhibit a similar resistance for all times investigated here.

### Oxygen migration in SrTiO_3_/SrO heterostructures

Given the significant retention improvement through the insertion of a SrO layer, we conclude that the Joule heating enabled phase separation into a switching element and an artificial retention-stabilization layer is not a side effect of high temperatures during forming. In fact, this phase separation is the origin of the observed stable retention for the device shown in Figs 1d–f and 2a,b. We therefore now consider how this phase separation can assist in prolongating the retention times. As discussed above, a retention failure is caused by the reoxidation of the switching filament from the vertical direction. For the SrTiO_3_/SrO heterostructure, three different scenarios can prevent this reoxidation:

- *Oxygen storage capability*. Similar to the oxygen exchange between different oxide layers in oxide dual-layer memory elements under applied electric fields^50^, oxygen from the SrTiO_3_ could be incorporated into SrO (as interstitials or into existing vacancies) during the SET operation. The SrO layer can be regarded as an oxygen reservoir. This may lead to a new (meta-)stable oxygen distribution between the two adjacent oxides eliminating the driving force for reoxidation of the SrTiO_3_ without applied electric fields and therefore lead to improved retention times.
- *Surface reaction rate of oxygen incorporation*. If the storage of additional oxygen in the retention-stabilization layer is impeded due to a lack of available lattice sites (no vacancies available and unfavourable energy of formation for oxygen interstitials), oxygen must be excorporated from the retention-stabilization layer during the SET operation, similar to the SET operation and reoxidation in SrTiO_3_ thin-film devices described above. In this case, reoxidation of the SrTiO_3_ during RESET or retention failure inevitably requires the incorporation and diffusion of additional oxygen into the heterostructure from the surroundings. Since conduction band electrons contribute to the rate-determining step in the incorporation of oxygen into the oxide lattice^51^,^52^, this step might be impeded in SrO due to the comparably large bandgap (between 5 and 6.5 eV). The inhibited oxygen incorporation may therefore be responsible for the enhanced retention times of the heterostructures.
- *Diffusion rate*. At the same time, the oxygen needs to migrate from the top surface into the conductive filament within the SrTiO_3_ after the incorporation into the retention-stabilization layer. Given the fast diffusion rates in SrTiO_3_, the improved retention of the heterostructures may be caused by slower ambipolar diffusion rates within the SrO. If the incorporation rate of the retention-stabilization layer was as fast as in SrTiO_3_, slow diffusion through the retention-stabilization layer may become the rate-determining step and thus retard the reoxidation of the switching filament.

**Figure 4:**
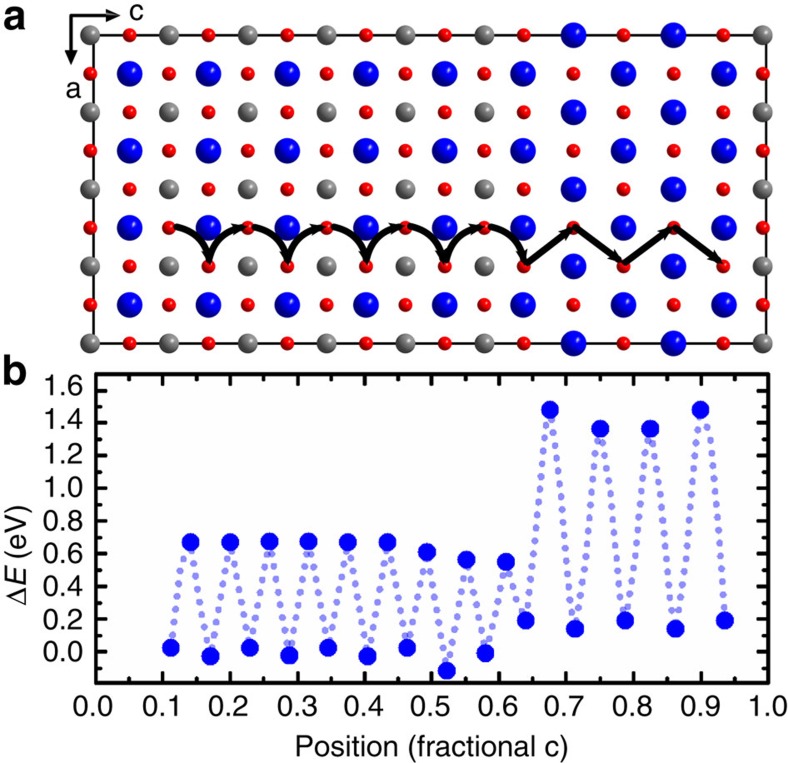
Oxygen-vacancy migration in the SrTiO_3_/SrO heterostructure. (**a**) Illustration of the simulation cell of the heterostructure, with the simulated migration path indicated schematically by black arrows. Sr in blue, Ti in grey and O in red. (**b**) Energies of the sites and saddle points corresponding to an oxygen vacancy migrating through the heterostructure. The *x* axis is scaled to the fractional coordinates of the *c* axis of the simulation cell. All plotted energies are referenced to the average vacancy formation in the SrTiO_3_ regime of the simulation cell. The dotted line serves as a guide to the eye for the change in energy during the migration process.

To clarify which of the effects (i–iii) are responsible for the improved retention times, we first describe the oxygen migration in the heterostructure through static lattice simulations. Given that both pulsed laser deposition and phase separation during electrical treatments result in oxide layers with considerable amounts of point defects such as anion and cation vacancies, we analysed the migration of oxygen vacancies within SrTiO_3_/SrO heterostructures. In Fig. 4, we show the site and saddle-point energies corresponding to the migration of an oxygen vacancy through the heterostructure. One notices a considerable difference in the migration barriers in the two materials, with the barriers in SrO (∼1.2 eV) being twice as high as those in SrTiO_3_ (∼0.6 eV). This suggests that, once oxygen ions are incorporated into SrO_1−*x*_ under an electric field (which lowers the barrier for migration), their return is hindered. There are also small differences in the site energies within SrTiO_3_ (this is due to the biaxial strain arising from lattice mismatch) and between SrTiO_3_ and SrO (suggesting a small amount of vacancy redistribution from SrO to SrTiO_3_)^52^.

On the basis of these calculations, we can conclude that impeded oxygen transport within the SrTiO_3_/SrO heterostructure (mechanism iii) could in fact lead to improved retention times. At the same time, we cannot exclude that mechanisms i and ii play a similar role in SrO, as oxygen incorporation reactions and the defect structure in SrO are not well understood. We therefore now turn to model oxide materials to clarify whether the oxygen storage capability or impeded oxygen transport is a dominating factor of retention improvement through the intentional insertion of retention-stabilization layers.

### Rational design of retention-failure-resistant memristive devices

The broad variety of physical properties exhibited by oxides allows us to select prototypical materials for the retention-stabilization layer. For this purpose, we select yttria-stabilized ZrO_2_ (YSZ) and Al_2_O_3_. These oxides differ most severely in two specific properties: electronically, both YSZ and Al_2_O_3_ are rather inactive insulators with a large bandgap, but they are prototypical representatives for fast oxygen transport combined with high oxygen-vacancy concentration and slow oxygen transport combined with very low oxygen-vacancy concentration, respectively. While YSZ is the prototypical oxygen-ion-conducting electrolyte, with a measured oxygen diffusion coefficient of *D*^YSZ^(500 K)≈10^−13^ cm^2^ s^−1^ and an activation enthalpy of just 1 eV (ref. 53), Al_2_O_3_ exhibits low diffusion coefficients, even close to its melting point; the extrapolated value with an activation energy of 6.5±0.5 eV (ref. 54) is 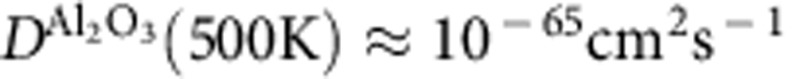. Due to the high concentration of oxygen vacancies, YSZ also exhibits a large amount of available lattice sites for oxygen storage, while Al_2_O_3_ exhibits very small deviations from nominal stoichiometry.

On the basis of these criteria, SrTiO_3_ (2 nm)/YSZ (1 nm) and SrTiO_3_ (2 nm)/Al_2_O_3_ (1 nm) heterostructures were fabricated by successive pulsed laser deposition. Multiple devices on each chip were switched between the HRS and the LRS using similar voltages as for the SrTiO_3_ thin-film devices. One may question whether the YSZ or Al_2_O_3_ layers might be actively involved in the resistive switching processes in these heterostructures. If the switching in these heterostructures was dominated by the inserted retention-stabilization layer, however, we would expect a different shape or even the opposite switching direction, as was demonstrated before for resistive switching in Al_2_O_3_ (ref. 55). Instead, we observed that the shape and polarity of the *I*–*V* characteristics as well as the absolute voltages and currents, which are typically observed in these heterostructures, were still the same as for SrTiO_3_ thin-film devices (Supplementary Fig. 3). Given that the resulting LRS state of the heterostructures is orders of magnitude smaller than the initial resistance or the HRS from the SrTiO_3_ thin-film devices, we conclude that the resistance change in the heterostructures must occur in the SrTiO_3_ layer, although we cannot rule out that additional processes take place in the YSZ or Al_2_O_3_ layers. Impedance spectroscopy experiments performed on virgin cells and cells in the LRS and HRS confirmed that the resistive switching in the heterostructures also occurs within a confined filament (Supplementary Fig. 4). For SrTiO_3_/YSZ heterostructures, we observe switching with a large initial memory window with a current compliance of 1 mA. For 10 mA current compliance, we observe a comparably low HRS of just 10^7^ Ω. Nevertheless, the initial memory window after switching with 10 mA current compliance is three orders of magnitude, which is similar to the memory windows obtained for the SrTiO_3_ thin-film devices. For even higher current compliances, we observe a device breakdown to a low resistance. SrTiO_3_/Al_2_O_3_ heterosturctures, on the other hand, can be switched reliably with 10 or 30 mA current compliance, resulting in similar initial memory windows of 10^3^ and 10^4^, respectively.

**Figure 5:**
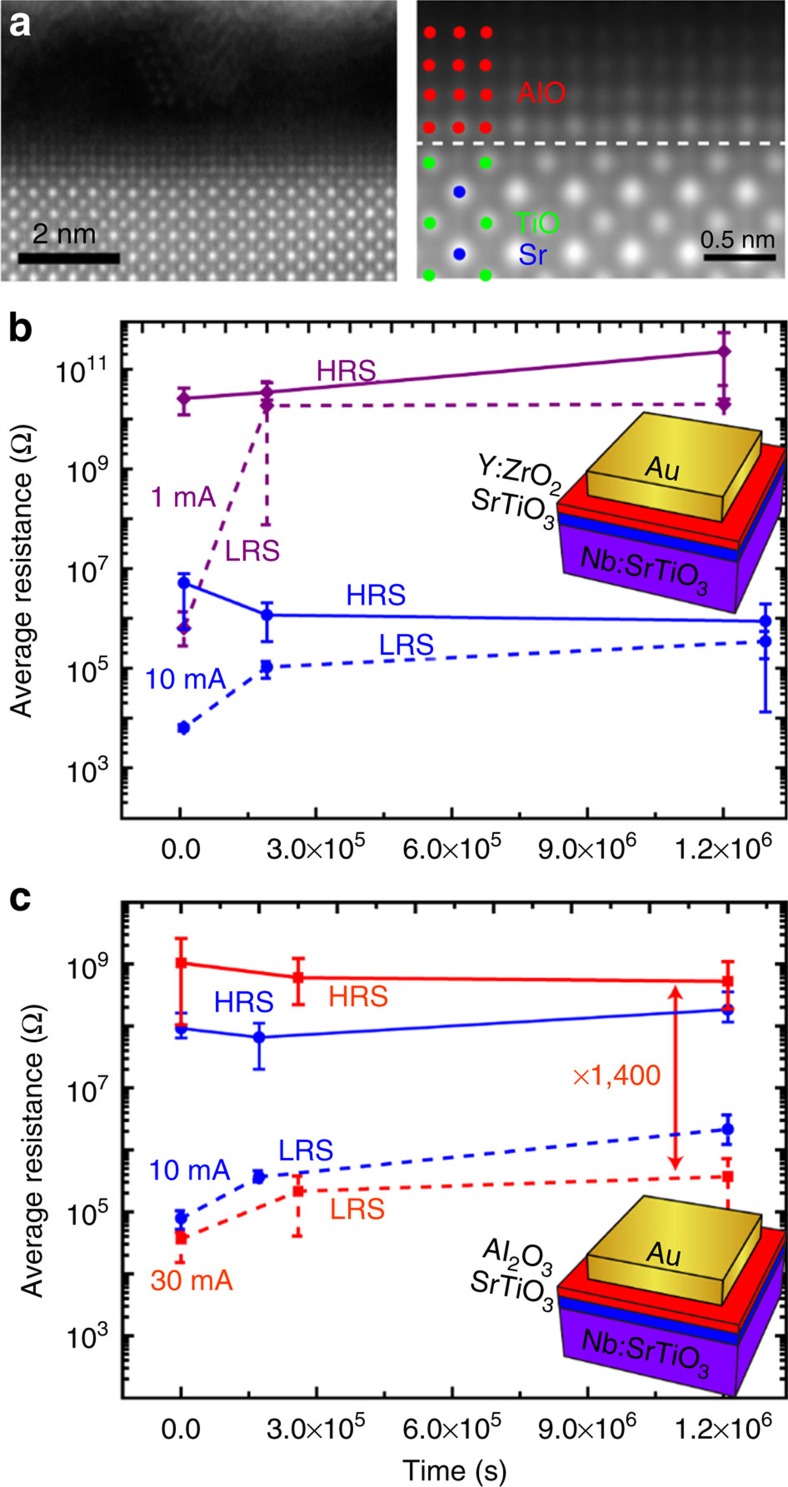
Retention behaviour of SrTiO_3_/YSZ heterostructures and SrTiO_3_/Al_2_O_3_ heterostructures. (**a**) Left: HAADF-STEM image of the epitaxial SrTiO_3_/Al_2_O_3_ interface (denoised by a nonlinear filter^62^); right: magnified one-dimensional averaged image better revealing the structure of the epitaxial interfaces. (**b**) Average resistance of SrTiO_3_/YSZ heterostructure devices in the LRS (dashed line) and the HRS (solid line) as a function of time after switching for a SET current compliance of 1 mA (violet lines) and 10 mA (blue lines). The device schematic is shown in the inset. (**c**) Average resistance of SrTiO_3_/Al_2_O_3_ heterostructure devices in the LRS (dashed line) and the HRS (solid line) as a function of time after switching for a SET current compliance of 10 mA (blue lines) and 30 mA (red lines). Error bars indicate the minimum and maximum values obtained for each resistance state. The device schematic is shown in the inset.

For closer inspection of the SrTiO_3_/Al_2_O_3_ heterostructure, we performed high-angle annular dark-field scanning transmission electron microscopy (HAADF-STEM) imaging of an electrically treated device, revealing epitaxial growth of the aluminium oxide on top of TiO-terminated SrTiO_3_. Figure 5a shows the cross-section HAADF-STEM images of the SrTiO_3_ (2 nm)/Al_2_O_3_ heterostructures from a switching filament, which was marked by conductive atomic force microscopy before the preparation of the TEM lamella (Supplementary Fig. 5). The observed atomic structure for the as-grown aluminium oxide is identical for the entire lamella (within the switching filament as well as few micrometres away from it) and appears to be similar to the reported SrTiO_3_/γ-Al_2_O_3_ in literature^56^,^57^. The TiO/AlO interfaces are more evidently seen from the one-dimensional averaged HAADF-STEM image.

On the basis of this structural characterization, we assume that our films are indeed good representatives for fast and slow oxygen transport coupled with high and low oxygen storage capabilities, respectively. Comparing the retention characteristics of these heterostructures, therefore, allows the distinction between mechanisms i and ii–iii. In fact, we observe compelling differences: HRS and LRS values were similar to each other after short times (<10^5^ s) for the case of the oxygen-conducting SrTiO_3_/YSZ heterostructure for all current compliances (Fig. 5b). For the oxygen-diffusion-suppressing Al_2_O_3_, on the other hand, stable retention of both HRS and LRS was obtained for both 10 and 30 mA, with a large memory window of several orders of magnitude even after more than 10^6^ s (as shown for the average resistance in Fig. 5c). As was the case for the SrTiO_3_/SrO heterostructures, we did not observe retention failure in any SrTiO_3_/Al_2_O_3_ device. This is a marked improvement compared with devices fabricated without retention-stabilization layers or retention-stabilization layers with fast oxygen diffusion. Since these heterostructures exhibit an initial memory window of 10^3^ for switching with 10 mA, which is nearly identical to the memory windows for SrTiO_3_/YSZ heterostructure or SrTiO_3_ thin-film devices, one can conclude that the observed stable retention cannot be explained by initially higher memory windows. We can now conclude, therefore, that inhibiting the back transport of oxygen (at room temperature and without applied electric fields) is the most effective method to stabilize retention in SrTiO_3_-based memristive devices. The oxygen storage capability (mechanism i) of the retention-stabilization layer does not appear to play an important role, as the introduction of YSZ layers does not improve retention. Instead, we must conclude from the observed effects that oxygen is excorporated from the oxide layers during the SET operation, and that the reoxidation of the switching filament can be prevented if no oxygen can enter the SrTiO_3_ (mechanisms ii and iii). As is evident from the improved retention with SrO and Al_2_O_3_ retention-stabilization layers, slow oxygen migration within the layer (mechanism iii) can protect the switching filament from fast reoxidation. Incorporation of such layers with slow oxygen diffusion for the protection of the switching filaments may therefore be regarded as a design rule for failure-resistant memristive devices. Since both SrO and Al_2_O_3_ are also wide-bandgap insulators, we cannot exclude that impeded incorporation of oxygen at the surface (mechanism ii) may have a similar or additional effect. However, in this case one may also expect a similar effect from YSZ, as the high initial resistance of SrTiO_3_/YSZ heterostructures (Supplementary Fig. 3) indicates that only an extremely small concentration of conduction band electrons is present in the YSZ layer. We conclude, therefore, that mechanism iii is the most apparent explanation for the improved retention.

Regarding the voltage–time dilemma described in the introduction, one might question whether the incorporation of such layers for retention stabilization through slow oxygen migration might not lead to very slow switching times simultaneously. The extreme nonlinearity of oxygen migration with respect to the applied electric field and temperature, however, can yield sufficiently fast switching times even for large oxygen-migration activation energies due to Joule heating^58^.

We would like to emphasize that only with Al_2_O_3_, the retention-stabilization layer with the highest oxygen-migration enthalpy used here, we were able to introduce stable retention with 10 mA current compliance. We concur that this current is still extremely high regarding the application in integrated circuits. However, the current compliances necessary to achieve resistive switching in micrometre-sized single-crystalline SrTiO_3_ devices are always on this order of magnitude^11^,^16^,^46^ and might be attributed to the inherently low defect density. To achieve stable retention in the SrTiO_3_-based devices shown in Figs 1 and 2, a much higher current compliance of 50 mA was applied, but the majority of devices still showed retention failure after short times. We conclude, therefore, that the intentional introduction of suitable retention-stabilization layers reduces the current necessary for the desired retention performance markedly.

This power reduction has very beneficial effects: major structural changes such as phase separations or severe lattice distortions are commonly observed for SrTiO_3_ thin-film devices (compare Fig. 2 and refs 9, 46). Within the switching filament shown in Fig. 5a, the structure in both the SrTiO_3_ and the Al_2_O_3_ shows no evident difference compared with the surrounding of the filament. The absence of structural changes of the active layers during switching is desirable for future memory and logic devices, yielding more uniform and reproducible devices. This is apparent from the much more uniform retention behaviour of SrTiO_3_/Al_2_O_3_ heterostructures (Fig. 5b) compared with the SrTiO_3_ thin-film devices in Fig. 1.

By purely empirical studies and without paying special attention to oxygen transport properties of the materials under consideration, several groups already observed improved device performance in memristive devices with inserted Al_2_O_3_ layers, such as improved uniformity of HfO_*x*_- and TaO_*x*_-based devices^59^,^60^, improved memory windows in TiO_*x*_-based devices^55^ and improved retention in GdO_*x*_-based devices^61^. On the basis of these observations, it can be assumed that our concept of the rationally designed retention-stabilization layer can be extrapolated from our model system to technologically more relevant systems.

In conclusion, we utilized the model system for valence-change-resistive switching, SrTiO_3_, to elucidate the impact of rationally designed retention-stabilization layers for stable retention in memristive devices. We demonstrated that spatially confined valence changes between Ti^3+^ and Ti^4+^ within a switching filament are responsible for the resistance change upon the application of electrical stimuli, as well as for the retention failure. While the low oxygen-migration enthalpy of SrTiO_3_ causes the frequently observed retention failure of the LRS, Joule-heating-induced local phase separation within the switching filament can stabilize the retention due to the formation of a SrTiO_3_/SrO interface, which inhibits fast reoxidation due to retarded oxygen transport through the SrO layer. We used this insight to derive a design rule for failure-resistant memristive devices: incorporation of oxide materials with slow oxygen diffusion, as the retention-stabilization layer protects the switching filaments from reoxidation and leads to significantly prolongated retention times, as was demonstrated here with SrO and Al_2_O_3_ layers. Utilizing good oxygen conductors such as YSZ as the retention-stabilization layer, on the other hand, yields frequent retention failure. In addition to improved retention, more uniform device performance can be achieved with Al_2_O_3_ retention-stabilization layers due to reduced heat generation compared with devices without intentional retention-stabilization layers.

## Methods

### Sample fabrication

Two-nanometre single-crystalline undoped SrTiO_3_ thin films were fabricated via pulsed laser deposition on 0.5-wt% Nb:SrTiO_3_ substrates (CrysTec). The single-crystalline SrTiO_3_ target was ablated by a KrF excimer laser (*λ*=248 nm) with a repetition rate of 5 Hz and a spot size of 2 mm^2^ at a target-to-substrate distance of 44 mm. The laser fluence was 1.4 J cm^−2^, yielding nearly stoichiometric films^49^. All samples were grown in an oxygen atmosphere of 0.1 mbar at a substrate temperature of 800 °C. The film growth was monitored using reflection high-energy electron diffraction (akSA400 system).

SrTiO_3_/SrO heterostructures were obtained by subsequent *in situ* pulsed laser deposition of 2 nm undoped, slightly Sr-deficient SrTiO_3_ thin films (laser fluence 1.75 J cm^−2^, oxygen atmosphere of 0.1 mbar) and 1 nm SrO (laser fluence 1.3 J cm^−2^, oxygen atmosphere of 10^−7^ mbar) on 0.5-wt% Nb:SrTiO_3_ substrates (CrysTec). A single-crystalline SrTiO_3_ and a polycrystalline SrO_2_ target were used, with a repetition rate of 1 Hz and a spot size of 2 mm^2^ at a target-to-substrate distance of 44 mm for both layers.

SrTiO_3_/YSZ and SrTiO_3_/Al_2_O_3_ heterostructures were obtained similarly. After SrTiO_3_ deposition as described above, the samples were cooled down to room temperature before *ex situ* pulsed laser deposition of YSZ or Al_2_O_3_ at room temperature. For both cases, the laser fluence was 2.1 J cm^−2^, with a repetition rate of 5 Hz and a spot size of 1.5 mm^2^ at a target-to-substrate distance of 60 mm in an oxygen atmosphere of 10^−4^ mbar.

For the top electrodes, a 30-nm Au layer was sputter deposited and structured into 10 × 10-μm^2^ electrode pads by optical lithography and reactive ion beam etching.

### Electrical characterization

For electrical characterization, the top electrodes were contacted with tungsten whisker probes. The Nb:SrTiO_3_ substrate served as an electrically grounded bottom electrode and was contacted by wire bonding. *I*–*V* sweeps were performed with a Keithley 2611A SourceMeter. The different sweeps were performed using the following voltage cycles: 0 V to positive voltages (maximum +3.75 V) for forming and SET, 0 V to negative voltages (maximum −5 V) for RESET and +0.5 V to −0.5 V for read-out. The device resistance was obtained from the slope of a linear fit of the read-out sweeps between −0.1 and +0.1 V. The step size was 20 mV and the holding time before measurement was 5 ms; the current compliance for the forming step and the SET process was between 1 and 50 mA, as indicated in the main text. During the RESET sweeps no current compliance was necessary. After the forming process, some of the cells were kept in the LRS and other were switched back into HRS for subsequent impedance spectroscopy and spectromicroscopic analysis. Impedance spectroscopy was performed on virgin cells and representative cells in the HRS and LRS using an HP 4291B RF Impedance/Material Analyzer with a sampling voltage of 300 mV and a frequency range from 20 to 10^6^ Hz. The impedance data were fitted with a commercially available software Zplot/Zview (Scibner Associates Inc.). An equivalent circuit consisting of a parallel circuit of a capacitor and a resistor was used to fit the data over the entire frequency range.

### Top electrode delamination

For spectromicroscopic analysis, the top electrodes were delaminated under ultra-high vacuum conditions with an adhesive copper tape after sputter deposition of a homogeneous 30 nm Au layer onto the entire sample. The delaminated sample was transferred into the XPEEM chamber under ultra-high vacuum conditions.

### Spectromicroscopy

All XPEEM experiments have been performed at the NanoESCA beamline at Elettra synchrotron laboratory (Trieste, Italy) using the endstation described elsewhere^62^. The complete removal of the Au top electrode is checked by recording of the spatially resolved Au 4f XPS spectrum on the former device area, and for all presented devices the absence of any gold signal has been verified (with the exception of a small amount of Au residue for the device presented in Supplementary Fig. 1). The total energy resolution determined from both the spectrometer broadening (the pass energy 50 eV and the entrance slit 1 mm) and the photon bandwidth was 200 meV for photoemission spectra. Various series of images were taken at increasing electron kinetic energies with a step size of 0.1 eV; the photon energy was 200 eV. Core level photoemission spectra were extracted from the resulting image stack in different regions of interest. Similarly, image stacks with increasing photon energy were recorded for absorption spectra, using a step size of 0.05 eV for the Ti L-edge and 0.2 eV for the O-K-edge. The electron energy above the Fermi edge *E−E*_F_ was 5.2 eV. The image stacks were analysed and spectra were extracted using the IGOR Pro software. The fitting of the core level photoelectron spectra was performed after principal component analysis with CasaXPS Version 2.3.16 (ref. 63). The components were constrained to have the same peak position and width for each fit. False colour maps for the Sr 3d core levels were created by fitting the spectra at each pixel using the peak model described in Fig. 2. For X-ray absorption, false colour maps were created by fitting the spectra at each pixel using model absorption spectra for Ti^3+^ and Ti^4+^, or O in SrO and O in SrTiO_3_, respectively. The colour scale represents the spectral contribution of the Ti^3+^ signal. For all image stacks, the linear, vertical energy dispersion of the beamline exit slit and the parabolic, vertical energy dispersion of the NanoESCA energy filter were corrected as described in ref. 64.

### TEM analysis

Cross-section TEM specimens were cut using focused ion beam milling with a Ga ion beam at 30 kV beam energy and polished at 5 kV on an FEI Helios NanoLab 400S, followed by thinning and cleaning in a Fischione 1040 Nanomill with an Ar ion beam at energy of 900 eV for thinning and 500 eV for cleaning, with filament current of 0.21 mA and specimen tilt angles of ±10°. HAADF-STEM imaging was conducted at 200 kV with an FEI Titan G2 80–200 ChemiSTEM microscope equipped with a high-brightness Schottky field emission electron gun, a probe *C*_S_ corrector and a Super-X EDX system. A nonlinear filtering algorithm was used for denoising of the HAADF-STEM images^65^. One-dimensional averaging of the HAADF-STEM images was carried out with a homemade package written in the Digital Micrograph scripting language.

### Computational methods

Static atomistic simulations of the SrTiO_3_/SrO heterostructure performed in this study employed empirical pair potentials of the form


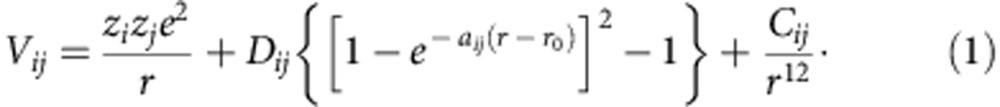


The first term is a coulomb term, which accounts for the long-range electrostatic interactions within the system. The second term is a Morse potential, which accounts for the short-range interactions, and the third term is a repulsive term, which corrects for Pauli repulsion and becomes important when performing high-temperature simulations. The empirical parameters of the potentials are obtained from the work of Pedone *et al.*^66^ and are listed in Table 1.

All degrees of freedom of the initial simulation cell of the SrTiO_3_/SrO heterostructure are optimized to a minimum energy configuration before the defect calculations. Defect calculations for determining vacancy and saddle-point configuration energies were performed employing the Mott–Littleton approach^67^. All simulations were implemented within the GULP code^68^.

## Additional information

**How to cite this article:** Baeumer, C. *et al.* Spectromicroscopic insights for rational design of redox-based memristive devices. *Nat. Commun.* 6:8610 doi: 10.1038/ncomms9610 (2015).

## Supplementary Material

Supplementary Information Supplementary Figures 1-5 and Supplementary Reference

## Figures and Tables

**Table 1:** Empirical parameters of the pair potentials used for the atomistic simulation.

	***D***_***ij***_**/eV**	***a***_***ij***_**/Å**^**−1**^	***r***_**0**_**/Å**	***C***_***ij***_**/eV Å**^**12**^
Sr^1.2+^–O^1.2−^	0.019623	1.886000	3.328330	3.0
Ti^2.4+^–O^1.2−^	0.024235	2.254703	2.708943	1.0
O^1.2−^–O^1.2−^	0.042395	1.379316	3.618701	22.0
