# Supplementary material for: Spectromicroscopic insights for rational design of redox-based memristive devices
Source: Nat Commun. 2015 Oct 19;6:8610. doi: 10.1038/ncomms9610 (PMC4634325; doi:10.1038/ncomms9610)
Supplement: Supplementary Information — Supplementary Figures 1-5 and Supplementary Reference [file ncomms9610-s1.pdf]

Supplementary Figures

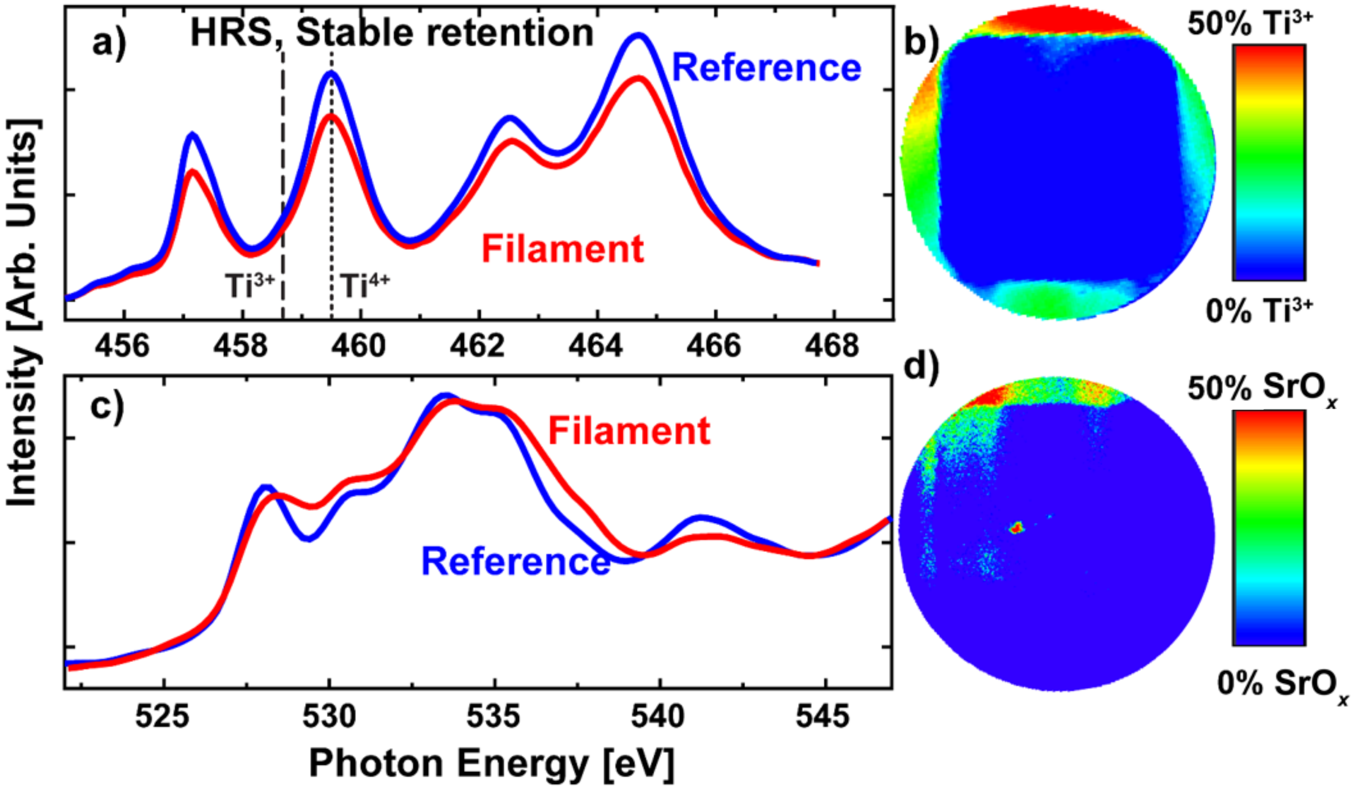

Supplementary Figure 1. Spectromicroscopic analysis of a device switched back into the HRS. a) Ti L-edge spectra extracted from the switching filament and from the surrounding device area, indicating no Ti^{3+} contribution. **b)** False color map of the Ti^{3+} contribution for the device in the HRS. **c)** O K-edge spectra extracted from the switching filament and from the surrounding device area, indicating significant SrO-contribution within the switching filament. **d)** False color map of the SrO_x-contribution for the device in the HRS. The presence of the phase separation discussed in the main text indicates that despite the phase separation, stable and reversible switching is possible.

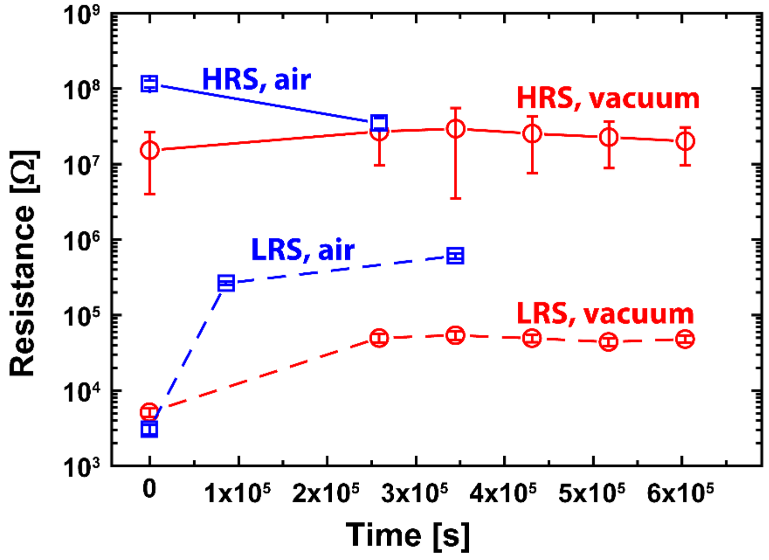

Supplementary Figure 2. Retention behavior of $SrTiO_3$ thin film devices in different atmospheres. Average resistance of $SrTiO_3$ thin film devices in the HRS (solid lines) and LRS (dashed lines) exposed to air (blue lines) or stored in vacuum (red lines). Measurements performed on a 20 nm $SrTiO_3$ film grown on a Nb:$SrTiO_3$ substrate *via* pulsed laser deposition. The substrate was used as bottom electrode. The devices were always switched in air with +/- 5 V supplied at a 10 nm Pt top electrode to switch them into LRS and HRS, respectively. The sample was then kept in air or in a vacuum chamber with a pressure of 10^{-6} mbar. Resistance values were obtained from the slope of a linear fit of the read-out sweeps between -0.1 V and +0.1 V and were averaged from 3 devices. Error bars indicate the standard deviation for each resistance state.

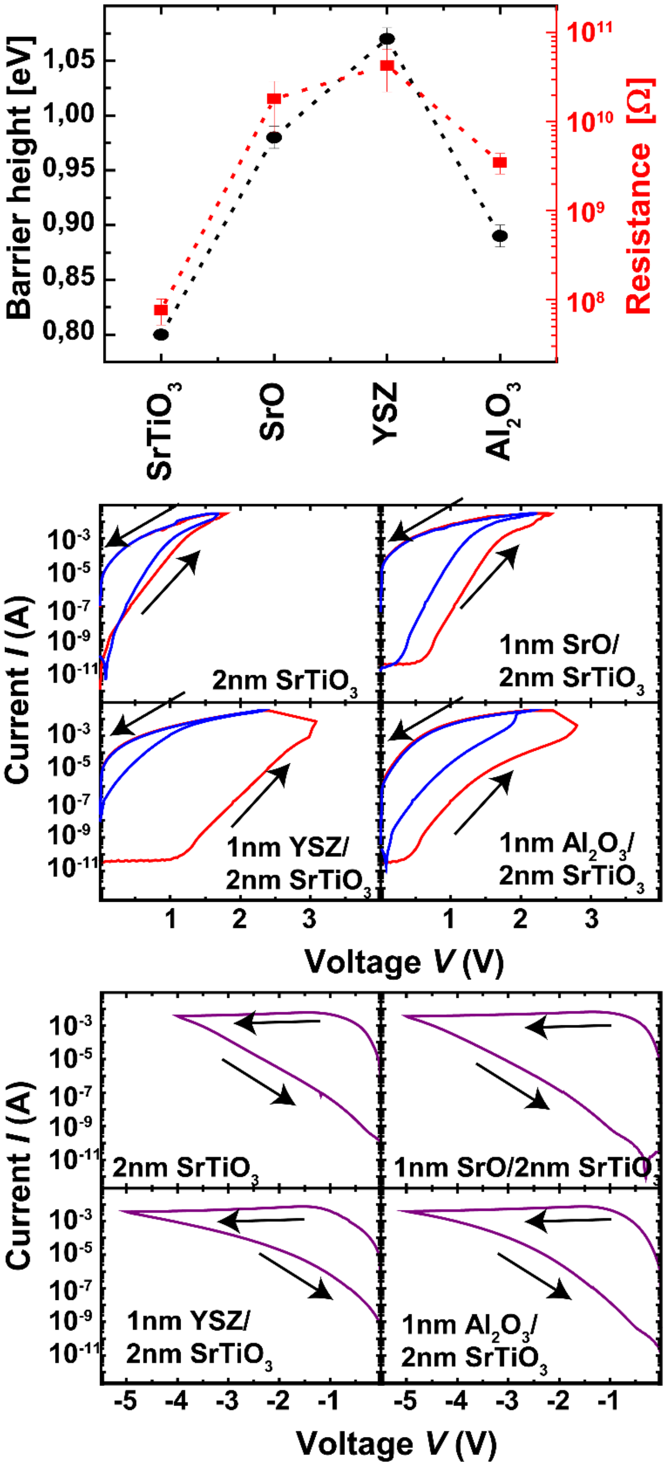

Supplementary Figure 3. *I-V* characteristics of oxide heterostructures. a) Initial resistance of the devices discussed in the main text and Schottky barrier height extracted from fits of the initial I(V)-curves in the reverse direction[1]. **b)** Forming step and SET operation for 2 nm stoichiometric $SrTiO_3$. **c)** Forming step and SET operation for $SrTiO_3$/ SrO heterostructures. Due to the higher initial resistance and the higher Schottky barrier, the forming step is more pronounced. The LRS value is very similar. **d)** Forming step and SET operation for $SrTiO_3$/ YSZ heterostructures. The forming is even more pronounced. **e)** Forming step and SET operation for $SrTiO_3$/ Al_2O_3 heterostructures. The current compliance was 30 mA in each case. After forming, the devices were RESET prior to the SET operation. **f)** RESET operation for 2 nm stoichiometric $SrTiO_3$. **g)** RESET operation for $SrTiO_3$/SrO heterostructures. **h)** RESET operation for $SrTiO_3$/ YSZ heterostructures. **k)** RESET operation for $SrTiO_3$/ Al_2O_3 heterostructures.

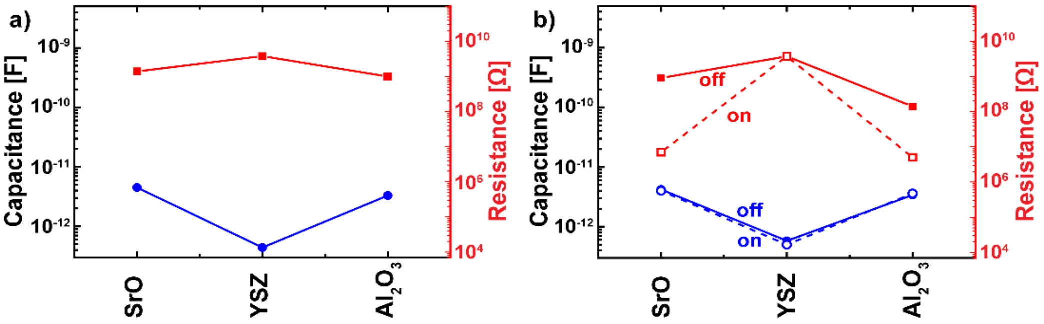

Supplementary Figure 4. Impedance spectroscopy results for different oxide heterostructures.
a) Capacitance (blue) and resistance (red) for each heterostructure discussed in the main text in the virgin state. **b)** Capacitance (blue) and resistance (red) for each heterostructure discussed in the main text in the HRS (solid lines) and LRS (dashed lines) after switching with 30 mA current compliance. The capacitance for each heterostructure does not change between virgin state, HRS and LRS, indicating filamentary switching. The resistance extracted from the impedance spectra indicates the retention behavior: for YSZ the HRS and the LRS are indistinguishable, while a sufficient memory window is obtained for SrO and Al_2O_3. Note that these measurements were performed on single devices and do not accurately describe the average resistance as demonstrated in the main text.

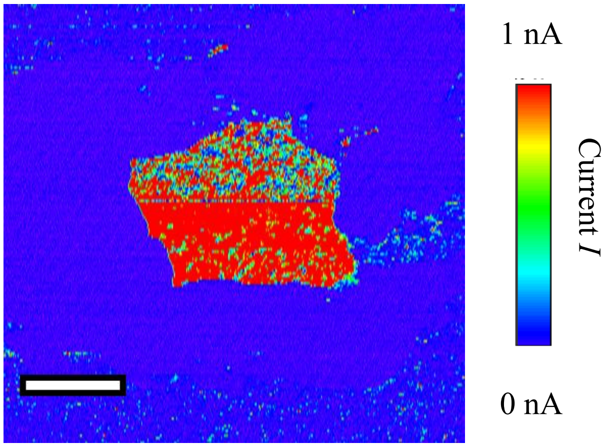

Supplementary Figure 5. Conductive AFM measurement of the switching filament of a $SrTiO_3/Al_2O_3$ heterostructure in the LRS. Scale bar is 500 nm. The gold top electrode of a $SrTiO_3/Al_2O_3$ heterostructure was delaminated and the switching filament was identified through conductive AFM with an Asylum Research Cypher AFM in air. A TEM lamella was prepared, which contained the conductive filament and the surrounding.

Supplementary References

1. Raab, N., Bäumer, C. & Dittmann, R. Impact of the cation-stoichiometry on the resistive switching and data retention of $SrTiO_3$ thin films. *AIP Adv.* **5,** 047150 (2015).
